# Surface quality and dry sliding wear behavior of AZ61Mg alloy using Abbott firestone technique

**DOI:** 10.1038/s41598-023-39413-x

**Published:** 2023-08-01

**Authors:** Eman H. El-Shenawy, Ahmed I. Z. Farahat

**Affiliations:** Plastic Deformation Department, Metal Technology Institute, Central Metallurgical R&D Institute (CMRDI), Cairo, 11421 Egypt

**Keywords:** Materials science, Theory and computation, Computational methods, Engineering, Mechanical engineering

## Abstract

Currently, magnesium alloys are widely utilized in diverse sectors due to their unique properties. However, the AZ61Mg alloy, a commonly used magnesium alloy, is known to have poor wear resistance, which limits its applications. To address this issue, researchers have investigated various surface treatment techniques, including the Abbott Firestone method, to improve the wear resistance of this alloy. This study employs response surface methodology (RSM) to examine the effects of pressure and velocity on wear behavior and Abbott Firestone zones of AZ61Mg alloy. Three pressure (0.01, 0.015, and 0.02 MPa) and velocity (0.57, 0.76, and 0.95 m/s) levels are used to conduct dry sliding wear tests at room temperature using a pin-on-disc method with an experimental design technique (EDT). Analysis of variance ANOVA is employed to identify the relationship between the input parameters (pressure and velocity) and the responses (wear rate, Surface Roughness Parameter Rz, and Abbott Firestone zones) of the AZ61Mg alloy. The optimized models for wear rate and Abbott Firestone zones yielded accurate estimations, which can enhance cost-effectiveness and efficiency. The findings indicate that pressure and velocity significantly affect the wear behavior of the AZ61Mg alloy.

## Introduction

Lightweight metals with excellent mechanical properties are currently being investigated as a potential solution to the energy crisis in the automotive and aerospace industries. Magnesium (Mg) is gaining popularity among researchers and scientists worldwide as one of the most promising lightweight metals. Magnesium is the lightest structural metal. Generally speaking, Mg has good qualities, such as high low density, damping capacity, and good size stability^1–3^. The hexagonal lattice structure of magnesium alloys highly affects their basic characteristics. Hexagonal lattice metals have more sophisticated plastic deformation than cubic lattice metals. Because the requirements for manufactured elements are constantly increasing, it is reasonable to strive to improve the quality of manufactured parts. Surface texture is the most commonly used indicator of surface quality^4^.

**Figure 1:**
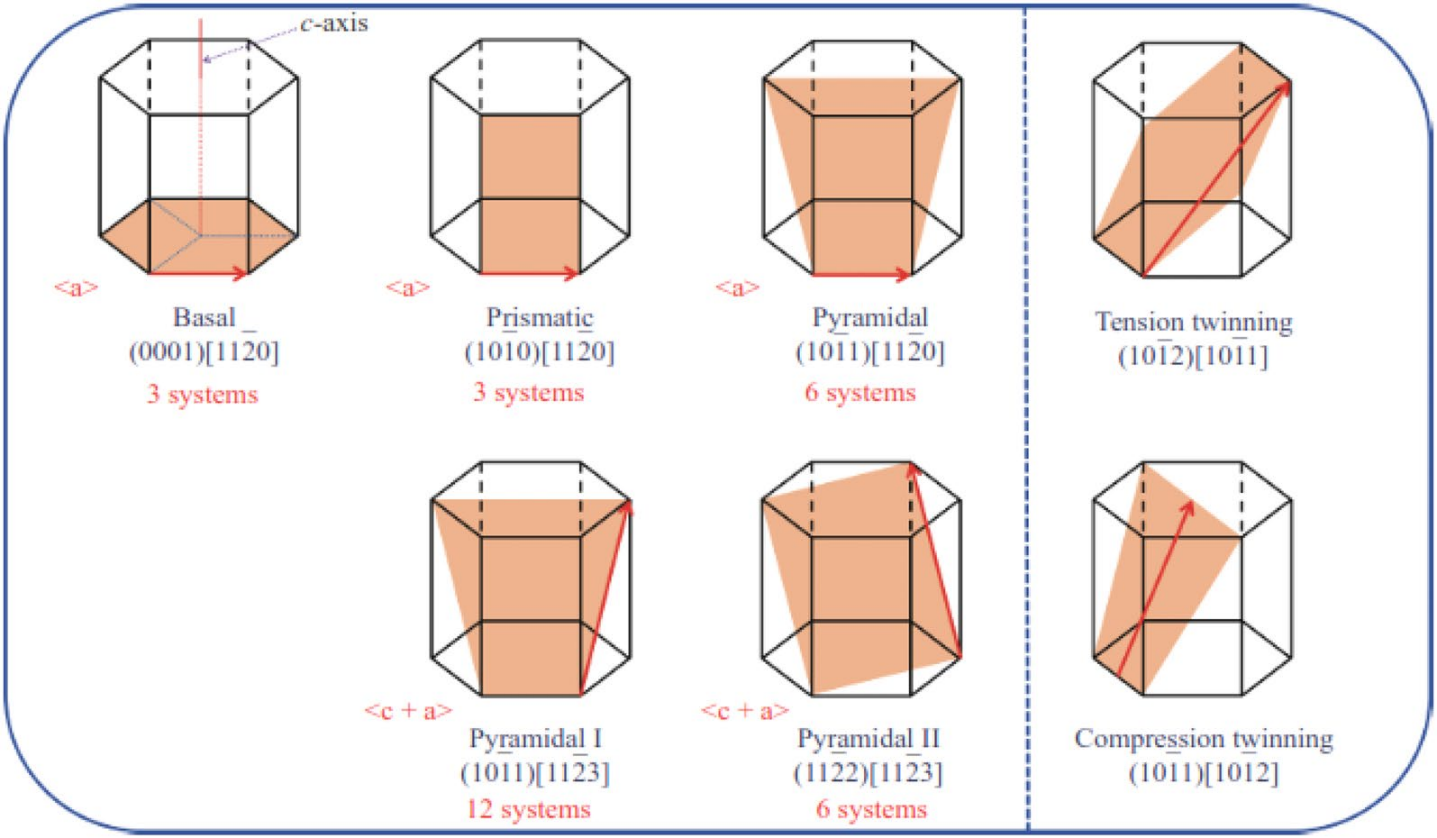
Common deformation modes in Mg: dislocation slips (left); twinning modes (right)^5^.

Figure 1 represents the most common deformation modes in Mg crystal structure, which include dislocation slip and twinning plans. In Mg, there are two types of slip systems: basal and non-basal slip systems (which include prismatic and pyramidal slip systems)^5^. Tension twinning (such as 1012 [1011]) and compression twinning (such as 1011 [1012]) are the most common twinning modes in Mg, accommodating tensile and compressive trains along the c-axis, respectively.

Although Mg has more slip systems than Al, its ductility is still lower, particularly at room temperature.

Although magnesium (Mg) alloys are the lightest structural metal, their hexagonal close-packed crystal structure makes them challenging to deform at low temperatures. To improve hot workability, non-basal slips can be activated at high temperatures during Mg alloy metalworking procedures^3,6^.

Surface texture is the most commonly used indicator of surface quality. However, scientific investigations have only examined the surface's 2D surface roughness metrics in order to assess its post-machining condition. Furthermore, Ra (arithmetic mean profile deviation) and Rz are the two most frequently utilized roughness parameters (a cusp height of the profile). For more assurance, a wider variety of 2D surface and 3D area roughness parameters ought to be given in the description^2^.

The Abbott Firestone curve is a tool that can be used to characterize the initial and worn surfaces of materials. It is more accurate than surface roughness (Ra) at capturing the changes that occur during wear. The curve can be used to assess the impact of synergistic processes, such as tribological ones, and to predict the likelihood of future changes to the surface^7,8^.

Sosa et al.^9,10^ used the Abbott Firestone curve to study the textural quality of gear teeth. They found that the voids in the surface appear to remain unaltered during wear, while the asperity peaks are worn off. Affatato et al.^11^ used the curve to identify the worn surface of a femoral head made of advanced ceramics.

Mathia and Pawlus^12^ emphasized the importance of surface characterization and testing when examining how different surface topographies affect tribological properties. Bruzzone et al.^13^ noted that the relationship between surface topography, function, and application is a particularly challenging undertaking that places a special emphasis on tribology.

Kara et al.^14^ investigated the effects of shallow and deep cryogenic treatment on Sleipner cold work tool steel in terms of microhardness, microstructure, coefficient of friction, and wear rate.

Design of experiments (DOE) and analysis of variance (ANOVA) techniques are frequently used in place of the time-consuming and expensive one-factor-at-a-time experimental technique. Response surface methodology (RSM) is a DOE technique that uses modeling techniques to establish the relationship between experiment input and output variables. RSM has been used to enhance process characteristics and to predict mechanical and tribological properties^15–18^.

Chauhan and Dass^19^ used RSM to investigate how load, speed, and sliding distance affected the wear resistance of titanium alloy (Grade 5). They found that the wear rate increases with an increase in the typical applied load and speed and drops with an increase in the sliding distance and a decrease in speed. Meddah et al.^20^ investigated the impact of load (P) and linear sliding speed (V) on the wear behavior and friction coefficient of 13Cr5Ni2Mo steel.

In the development of stressed parts, such as medical implants, it is crucial for surfaces to function consistently and display specific functional attributes, such as fatigue strength, tribological properties, and adhesive properties. The prediction of these characteristics is essential in the context of sliding friction phenomena and abrasive wear.

Although magnesium alloys have become increasingly prevalent in various fields, such as space, medical implants, and automotive industries, they suffer from low ductility at room temperature. Wear characteristics studies have shown that AZ61Mg alloy has a relatively high wear rate, especially under high loads and sliding speeds, which can lead to premature failure of components like gears. Therefore, it is necessary to investigate the wear behavior of AZ61Mg alloy using a reliable and quantitative technique like the Abbott Firestone technique to evaluate the worn surface and identify the most crucial exploitation zone to prevent gear failure.

By addressing the shortcomings of AZ61Mg alloy, particularly its low wear resistance, this study could contribute to the wider use of magnesium alloys in various industrial applications, such as aerospace, automotive, and medical implants.

## Experimental work

The experimental material was cast AZ61 Mg alloy with a chemical composition of Mg–6.14Al–1.39Zn–0.15Si-0.13Cu (wt%) and hot rolled to dissolve the complicated phase (Mg17Al12 precipitates) in a matrix. The phases that possibly form in the AZ61 Mg alloy under nonequilibrium and equilibrium conditions were calculated using thermodynamic phase diagram software JMatPro, which was linked with a thermodynamic database for magnesium alloys.

The present study conducted a wear test on cylindrical wear specimens using a pin-on-ring tribometer testing apparatus under dry conditions at ambient temperature. The wear experiments were performed in triplicate, and the average wear rate was calculated. The wearing tool used in the experiments was a spinning hardened stainless-steel ring with an outer diameter of 73 mm and a surface hardness of 63 HRC. The wear specimens had a cylindrical shape with a diameter of 6 mm and a length of 15 mm. Prior to each test, the ring surface was polished using various emery sheets with a grit size of 1000. A constant applied load of 50 N was applied for 5 min, with three different pressures (0.01, 0.015, and 0.02 MPa) and various linear sliding speeds (0.57, 0.76, and 0.95 m/s) being employed. The surface roughness of the top circular base of the printed samples was measured using the Mitutoyo Surftest SJ-201.

Before the wear testing, the weight of the samples was determined using an electronic scale with a precision of 0.1 mg. The worn surfaces of the wear-tested specimens were analyzed using field emission scanning electron microscopy (FESEM). The worn surface photographs were processed analytically and graphically using Gwyddion and Matlab software. Statistical analysis and Excel software were used to produce surface roughness and Abbott Firestone curves.

Wear Experiments were carried out at different pressures in MPa and linear velocities in m/s, as listed in Table 1.

**Table 1:** Different levels of wear pressure, linear velocities, and surface roughness characteristics.

Experiment no	P (MPa)	V (m/s)	Ra (Avg.)	Ry (Avg.)	Rz (Avg.)	Rq (Avg.)
1	0.01	0.57	4.35	26.63	26.63	5.72
2	0.01	0.76	3.33	22.12	22.12	4.56
3	0.01	0.95	4.00	20.71	20.71	4.87
4	0.015	0.57	3.42	20.08	20.08	4.46
5	0.015	0.76	3.67	21.17	21.17	4.73
6	0.015	0.95	2.84	16.32	16.32	3.55
7	0.02	0.57	2.60	14.45	14.45	3.25
8	0.02	0.76	2.51	14.53	14.53	3.23
9	0.02	0.95	3.13	18.16	18.16	4.04

## Results and discussions

**Figure 2:**
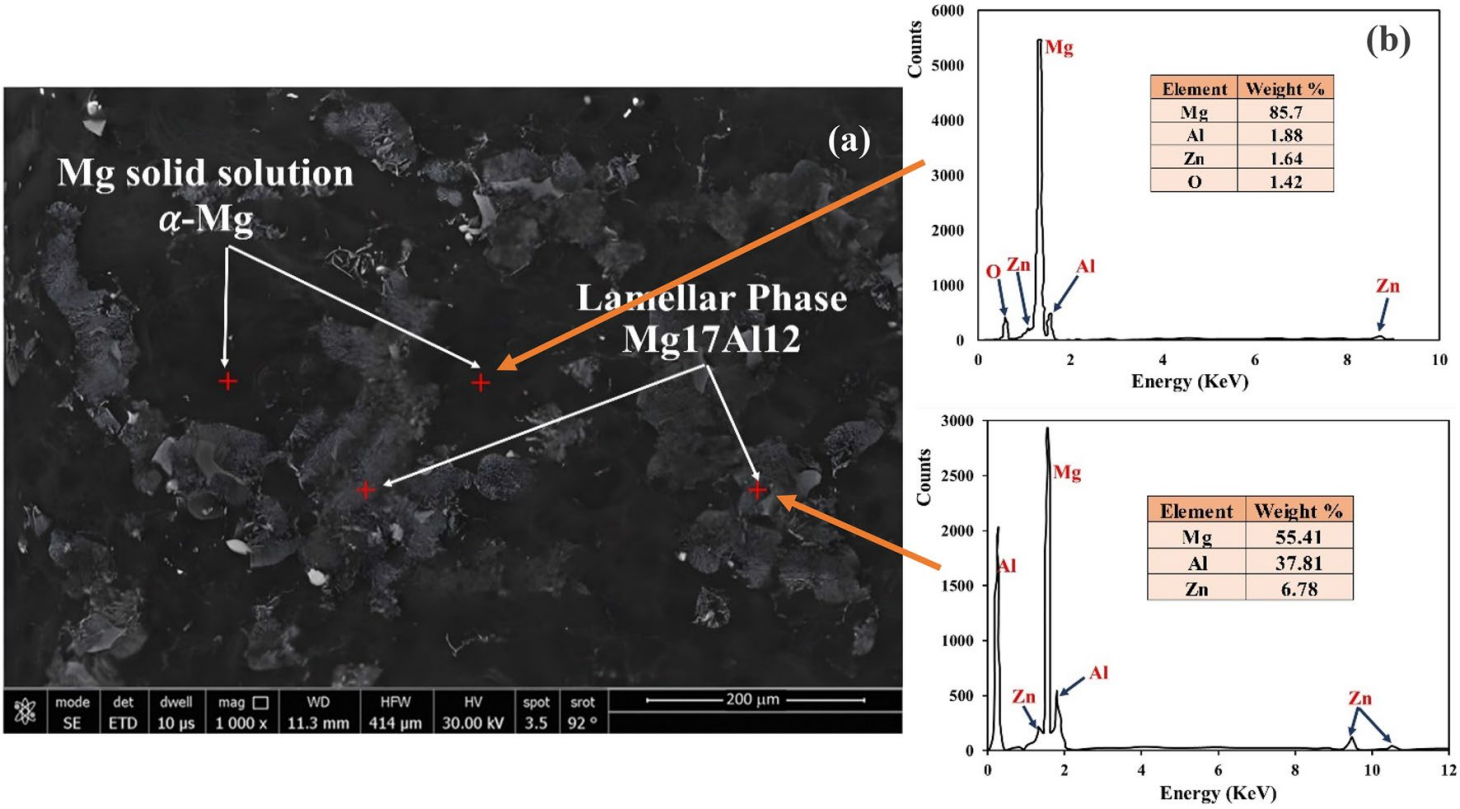
Microstructure (**a**) and EDS (**b**) of as-received Mg alloy AZ61.

The SEM image in Fig. 2 shows an AZ61 alloy specimen that has not been heat-treated. The specimen consists of a combination of two materials—a depleted magnesium solid solution known as α-Mg, and an intermetallic compound called Mg17Al12. The Mg17Al12 compound appears as a mixture of both continuous and discontinuous β-phases at the grain boundaries. The EDS analysis of the specimen in Fig. 2b reveals that both the bright and dark phases in the image contain magnesium, zinc, oxygen, and aluminum elements.

**Figure 3:**
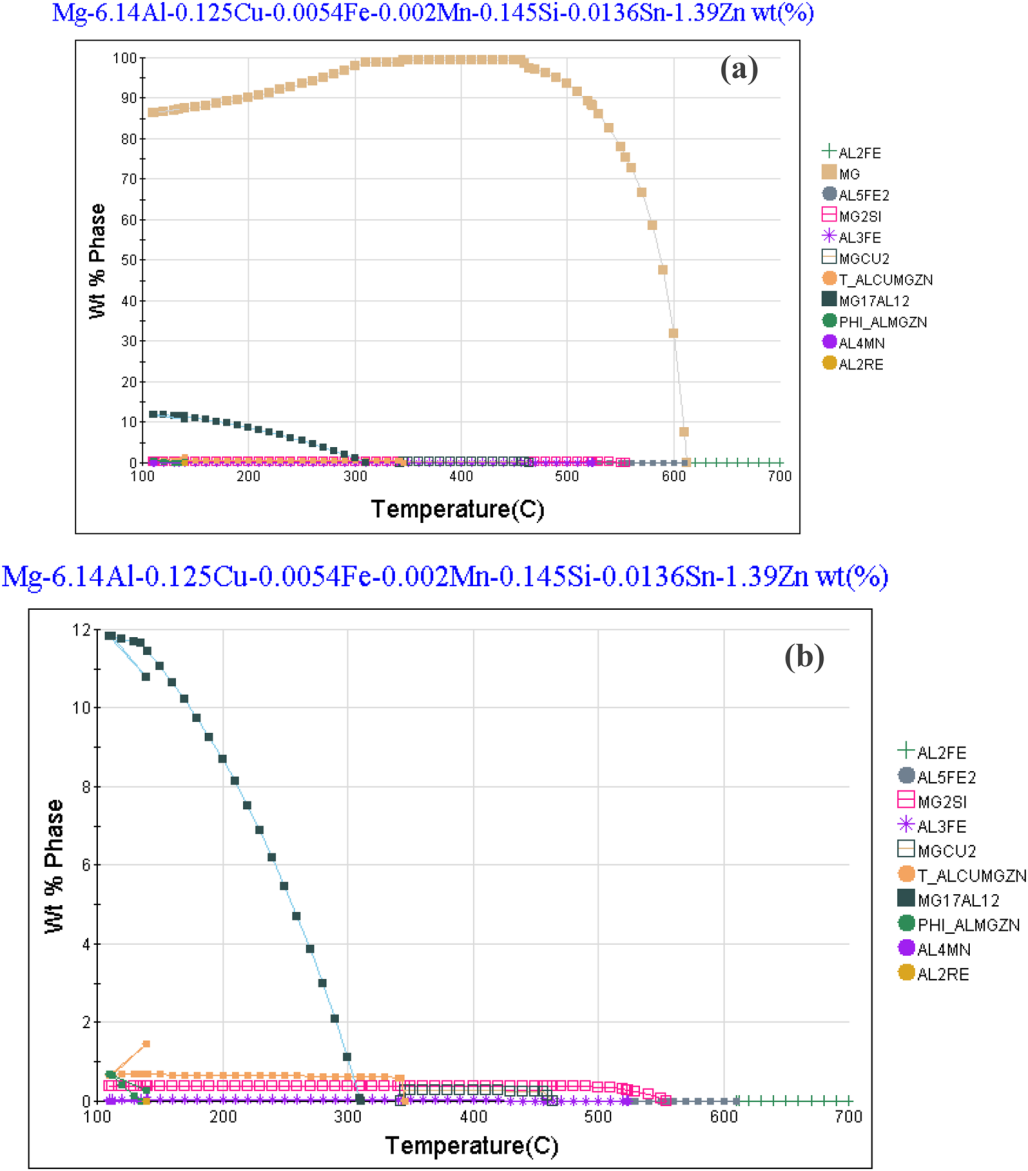
Types of phases and their phase fraction in the AZ61 Mg alloy calculated using the equilibrium and models.

It consists of mainly Mg matrix containing Mg17Al12 islands (brittle phase). This is emphasized by JMatPro software, where the volume fraction of Mg (alpha phase) is more than 85%, as seen in Fig. 3 below. The volume fraction of Mg17Al12 islands is about 12%.

The equilibrium model was used to calculate the types and amounts of phases present in an as-received AZ61 Mg alloy based on its chemical composition, and the results are shown in Fig. 2a,b. According to the model, the main secondary phase in the alloy is Mg17Al12, which makes up around 12% of its weight. The microstructure also contains small amounts of T_AlCuMgZn, Mg2Si, and Al4Mn. The model predicts that these minor phases, which are present in the interdendritic area of the as-cast microstructure, will disappear during homogenization treatment and re-precipitate during slow cooling after homogenization. XRD analysis of the alloy, shown in Fig. 3, confirms the presence of two types of compounds in the microstructure—α-Mg as the matrix and Mg17Al12 as the primary, secondary phase.

**Figure 4:**
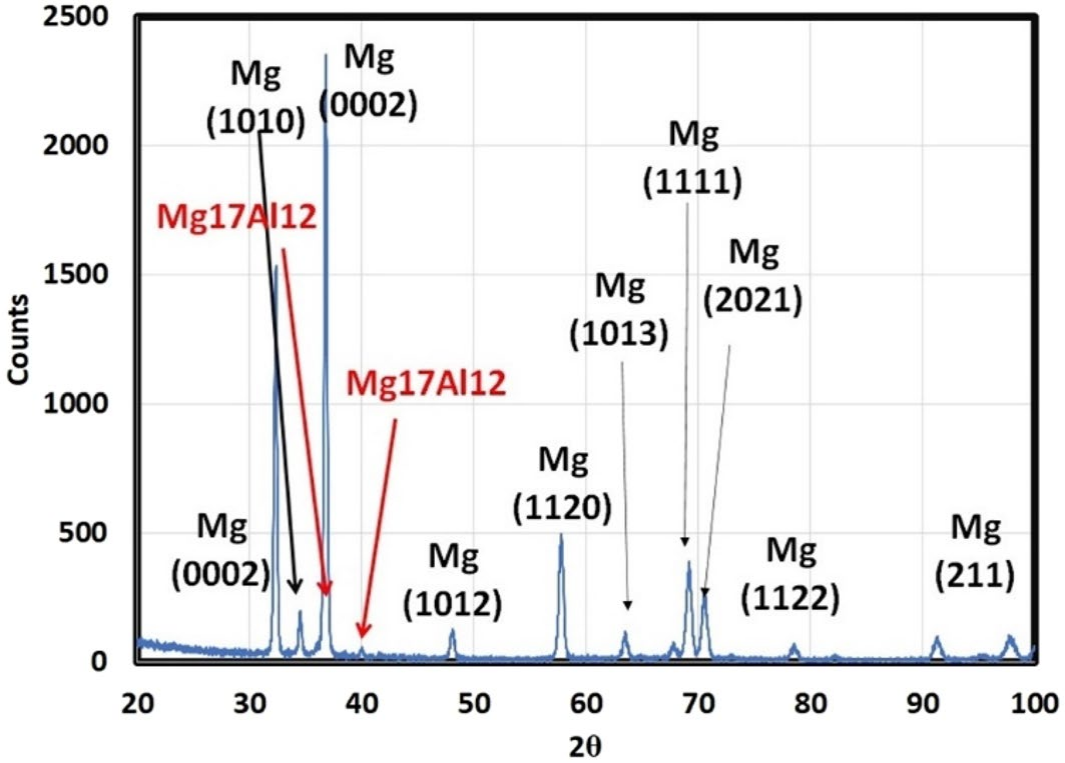
XRD of AZ61.

Figure 4 is the XRD plateau for AZ61. It shows precipitates of Mg17Al12 complex. Also, it shows that the basal plane is a prominent peak (0001).

The preceding figures offer a comprehensive depiction of the microstructural features and elemental composition of an AZ61 magnesium alloy specimen, detailing the distribution of various phases and minor components within the microstructure, utilizing advanced analytical tools such as FESEM, EDS, XRD, and JMatPro software.

**Figure 5:**
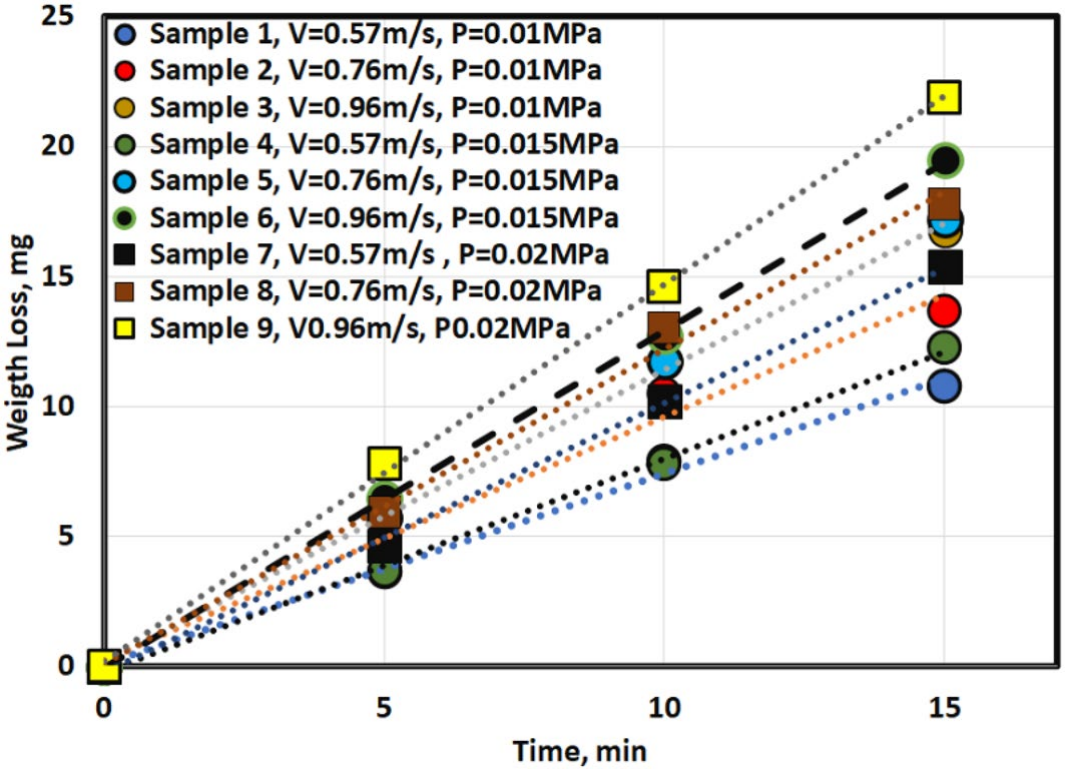
Weight loss at different velocities and pressure.

Figure 5 shows the weight loss of AZ61 Mg alloy after 15 min at different pressures (0.01–0.02 MPa) and velocities (0.57–0.96 m/s).

The results presented in Fig. 5 reveal a clear relationship between the duration of wear testing in minutes and the corresponding weight loss in milligrams under different pressures in MPa and velocities in m/sec. The data shows that the weight loss of the material increases as the duration of the wear test is prolonged. These findings suggest that prolonged exposure to wear can lead to the progressive degradation of the material.

The wear behavior of the tested material was evaluated under varying pressure and velocity conditions, with the highest weight loss observed at maximum pressure (0.02 MPa) and velocity (0.96 m/s), while the lowest weight loss was observed at medium pressure (0.01 MPa) and velocity (0.57 m/s). However, the effect of pressure and velocity on the wear rate could not be clearly distinguished from Fig. 5, emphasizing the need for a comprehensive study of both parameters. To this end, a mathematical model was constructed to express the wear rate as a function of pressure and velocity, and an Analysis of Variance (ANOVA) was employed to analyze the wear rate behavior due to the two parameters.

**Figure 6:**
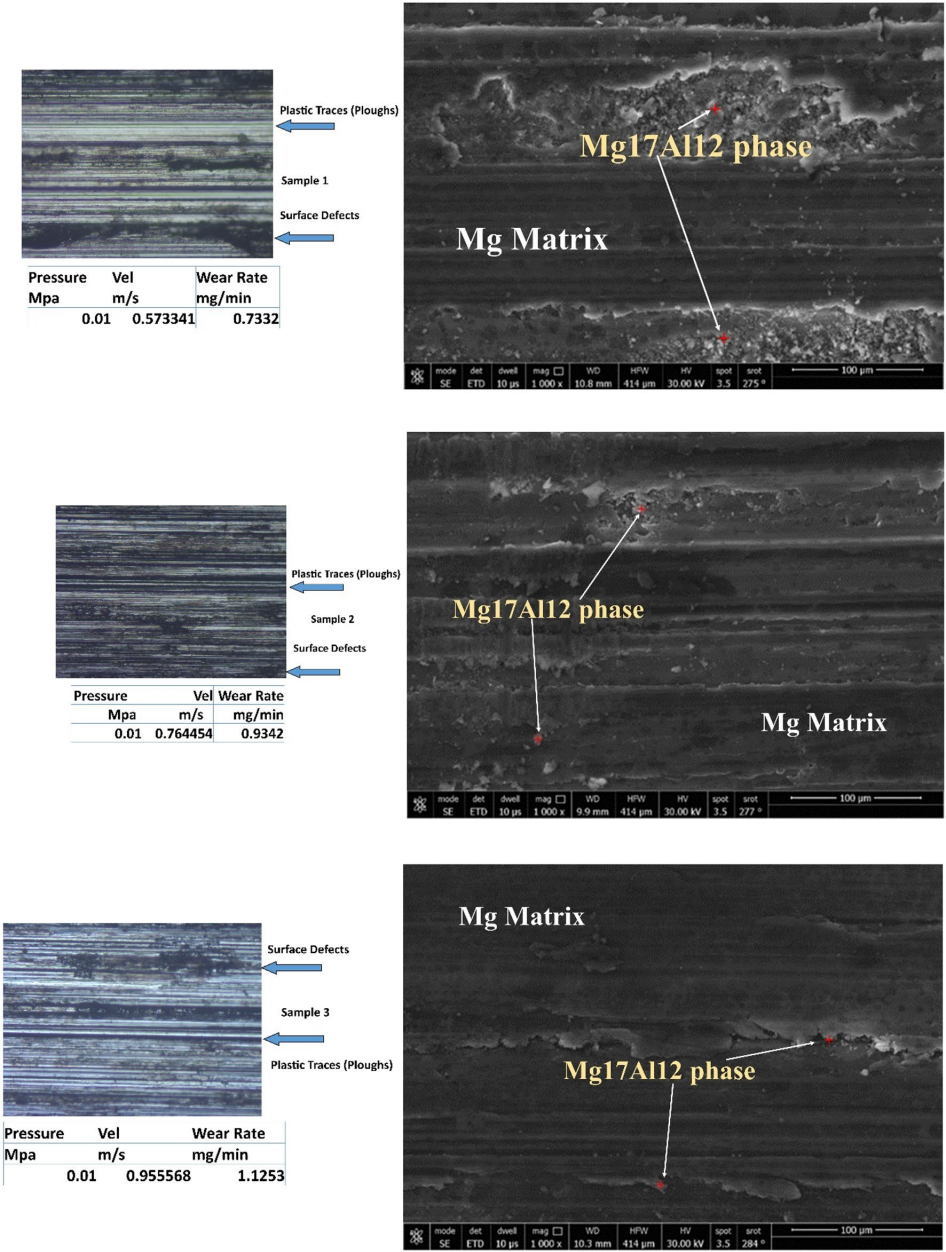

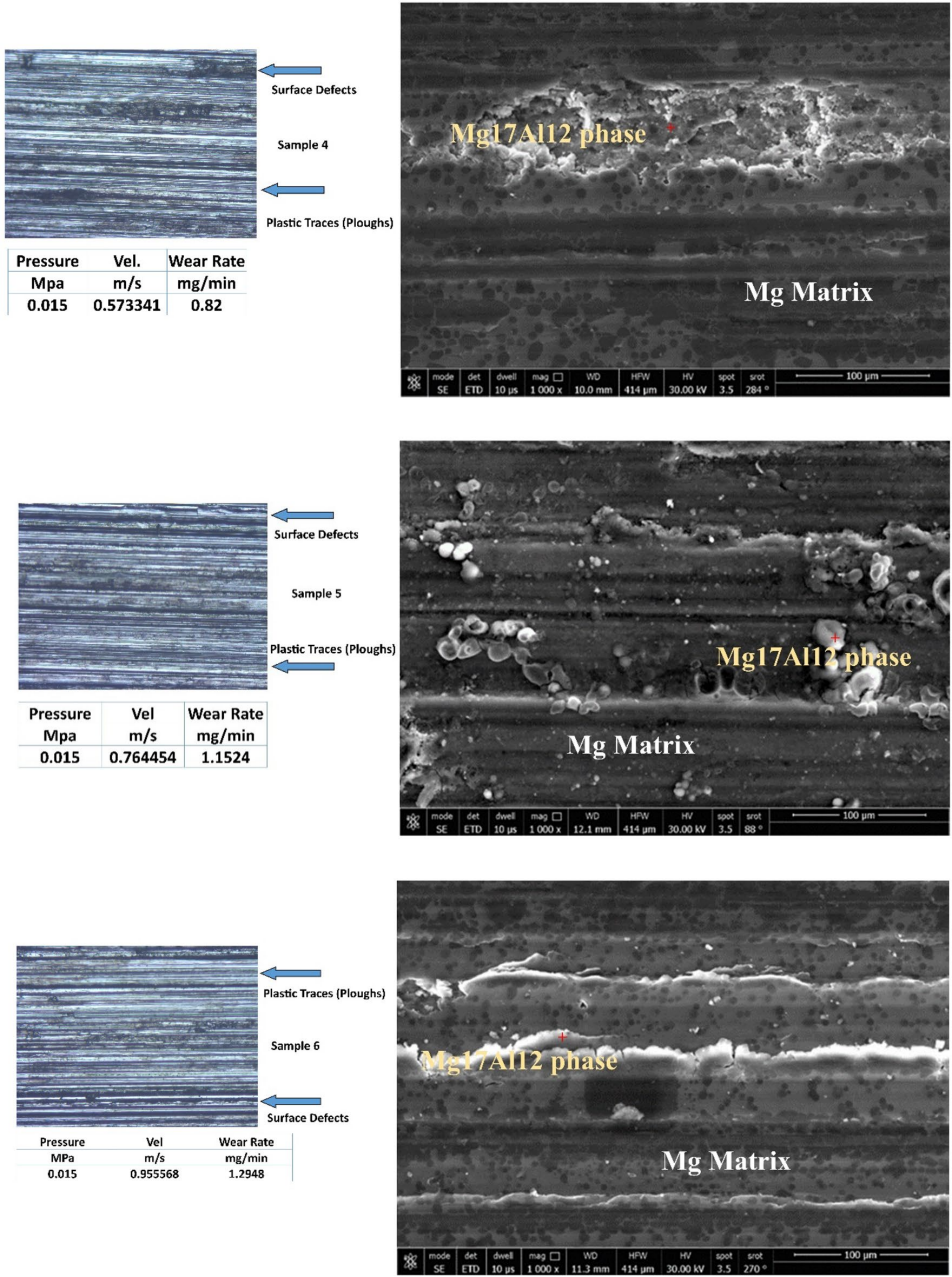

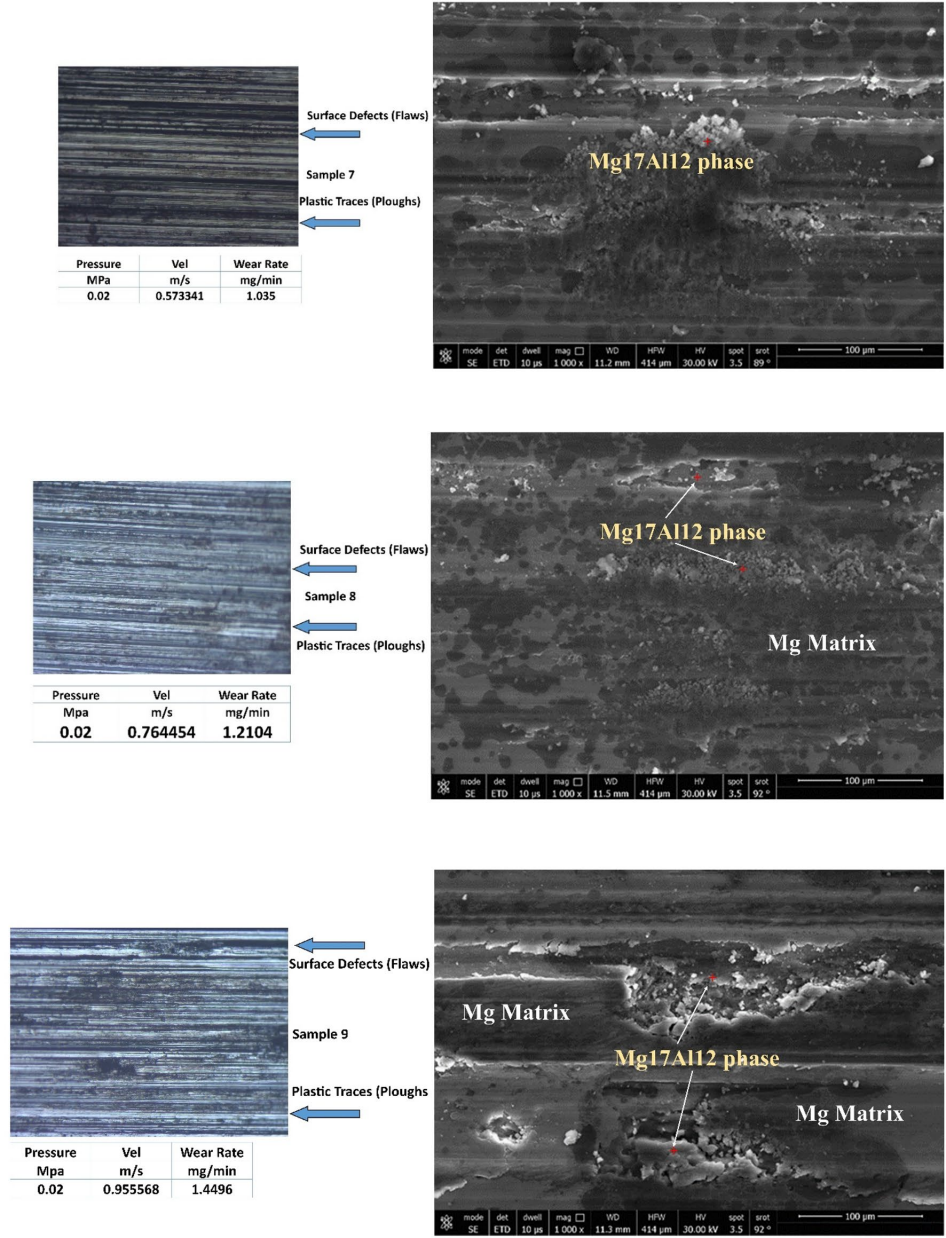
Optical and FESEM worn surface details of different samples.

Figure 6 represents worn surfaces at different conditions (such as velocities and pressures). Some optical photos, like samples 1 and 2, exhibit deep and dark pits (as seen in optical images). It might be due to Al12Zn17 complex precipitates, which make three bodies abrasive wear mechanisms. FESEM photos emphasize the existence of Al12Zn17 complex precipitates. All samples under all tribological parameters (velocities and pressures) exhibit plastic lines (Ploughs) due to adhesive wear mechanism. The friction types are abrasion, adhesion, and ploughing.

**Figure 7:**
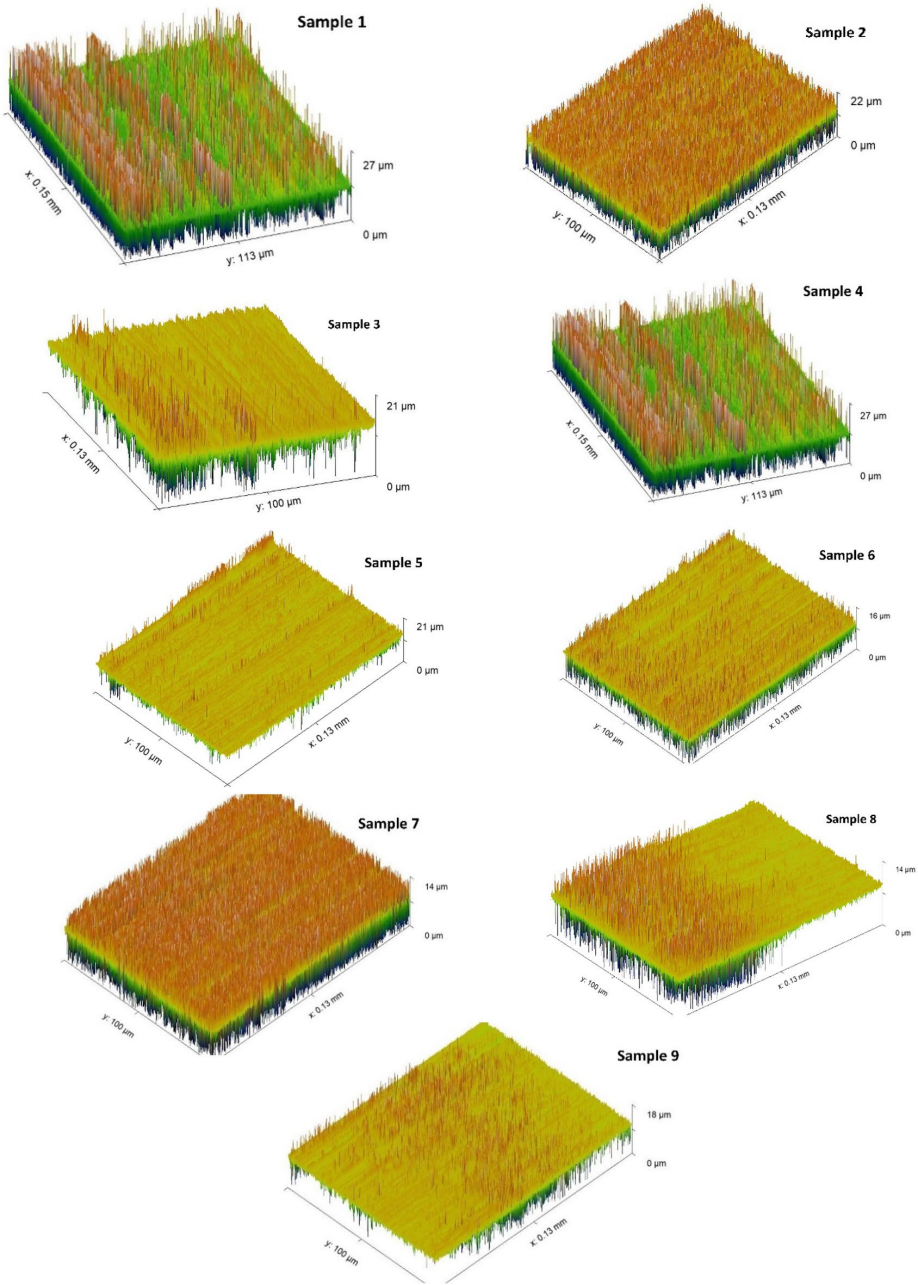
3D worn surface of different samples.

Figure 7 describes the 3D worn surfaces. It is noticed that Sample 2 and 7 only produces homogeneous worn surface; however, samples 1, 3, 4, 5, 6, 8, and 9 has an inhomogeneous worn surface.

**Figure 8:**
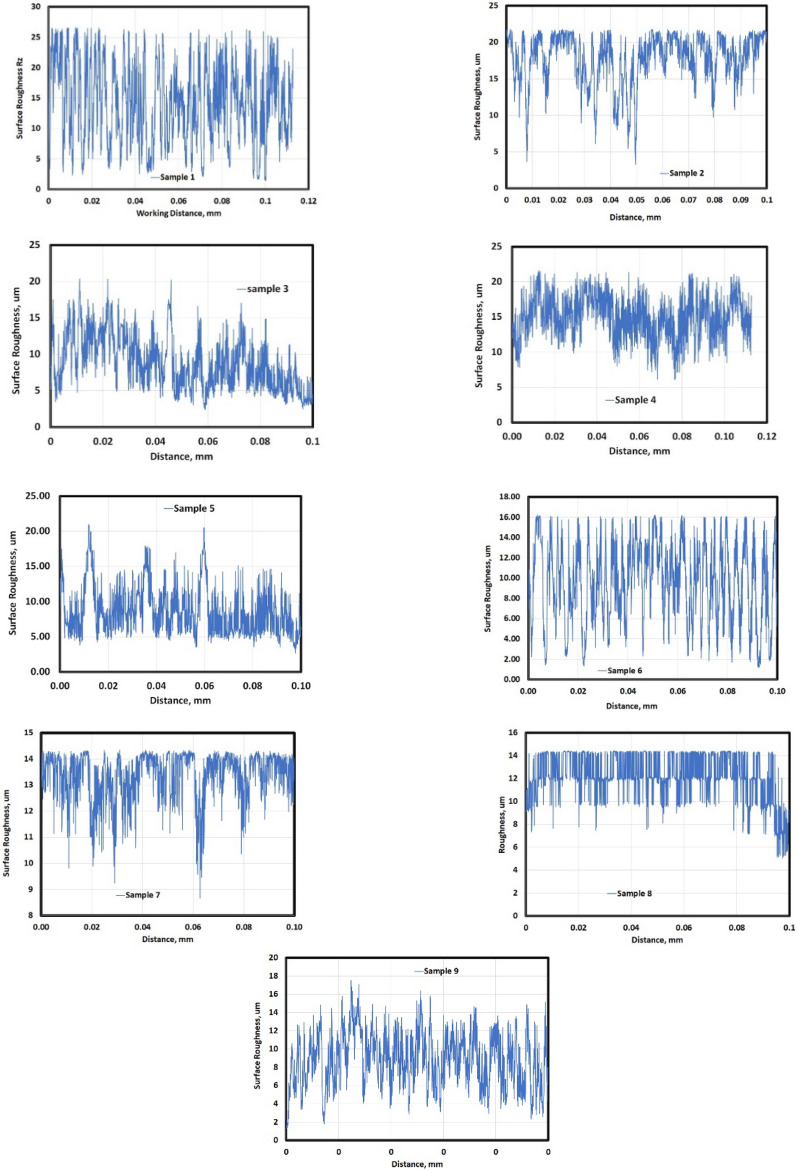
Profile of worn surface of different samples.

Figure 8 describes the different average peaks (Rz) for other tribological conditions. Samples 3, 7, and 8 exhibit wide peaks of contact surface, while the rest of samples have narrow peaks (points) of contact surface.

**Figure 9:**
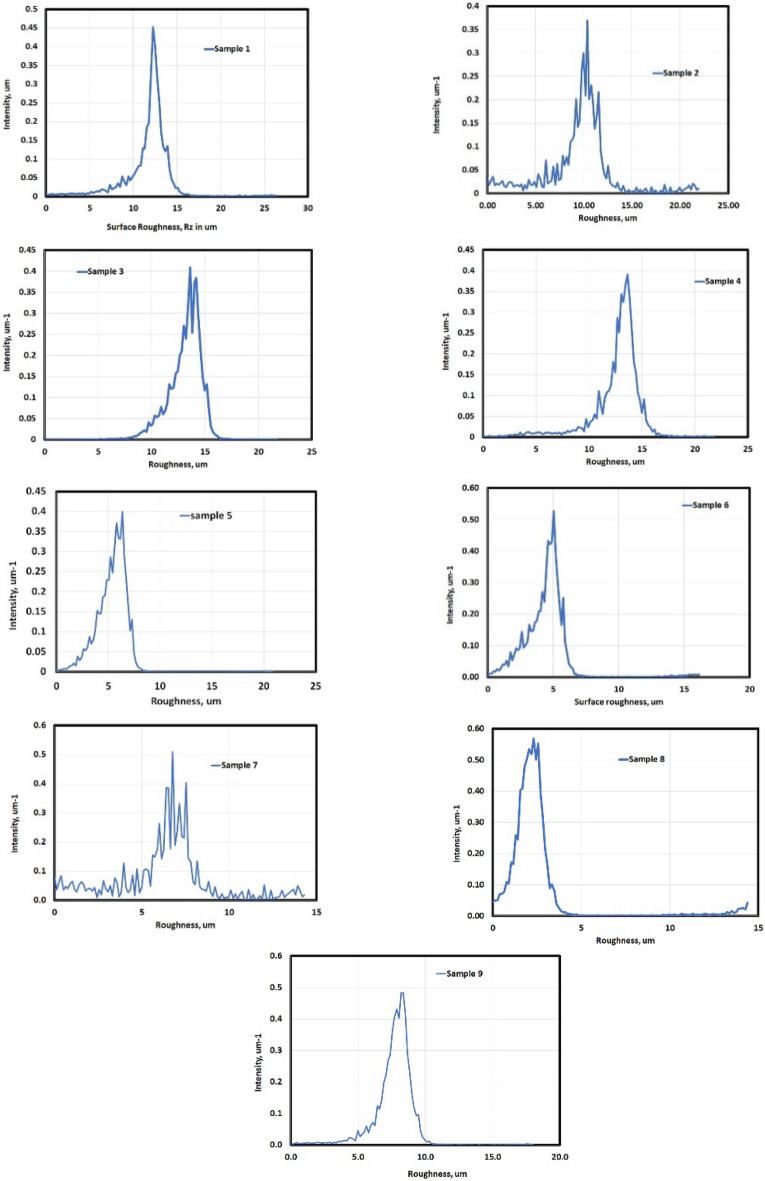
Normal distribution of surface roughness for different samples.

Figure 9 illustrates the distribution intensity of worn surface flaws. Samples 2, 3, and 7 exhibit several worn surface flaws due to three bodies mechanism.

The surface roughness of the worn specimens was evaluated using three figures. Figure 7 shows that the surface roughness profiles varied significantly between the samples. Only Samples 2 and 7 had homogeneous worn surfaces, while the others were inhomogeneous.

Figure 8 shows the average surface roughness values calculated using MATLAB software. It reveals the different average peak heights (Rz) for the test conditions. Samples 3, 7, and 8 had wider peaks indicating rougher contact surfaces, while the others had narrower peaks. This matches the different profiles in Fig. 7.

The average surface roughness generally increased with sliding velocity for all conditions. However, these profiles could not quantify the texture in detail. The Abbott Firestone technique was used to quantify the surface roughness due to different velocities and pressures.

Figure 9 illustrates the intensity of worn surface flaws. Samples 2, 3, and 7 showed several flaws likely due to three-body wear.

Mathematical modeling that simulates the wear rate versus velocity and pressure was constructed to better understand the worn surface behavior and determine the key parameter (pressure or velocity). It is crucial to investigate how Abbott Firestone zones relate to pressure and velocity and build a model expressing their relationships to quantify the effects.

### Abbott Firestone curves

**Figure 10:**
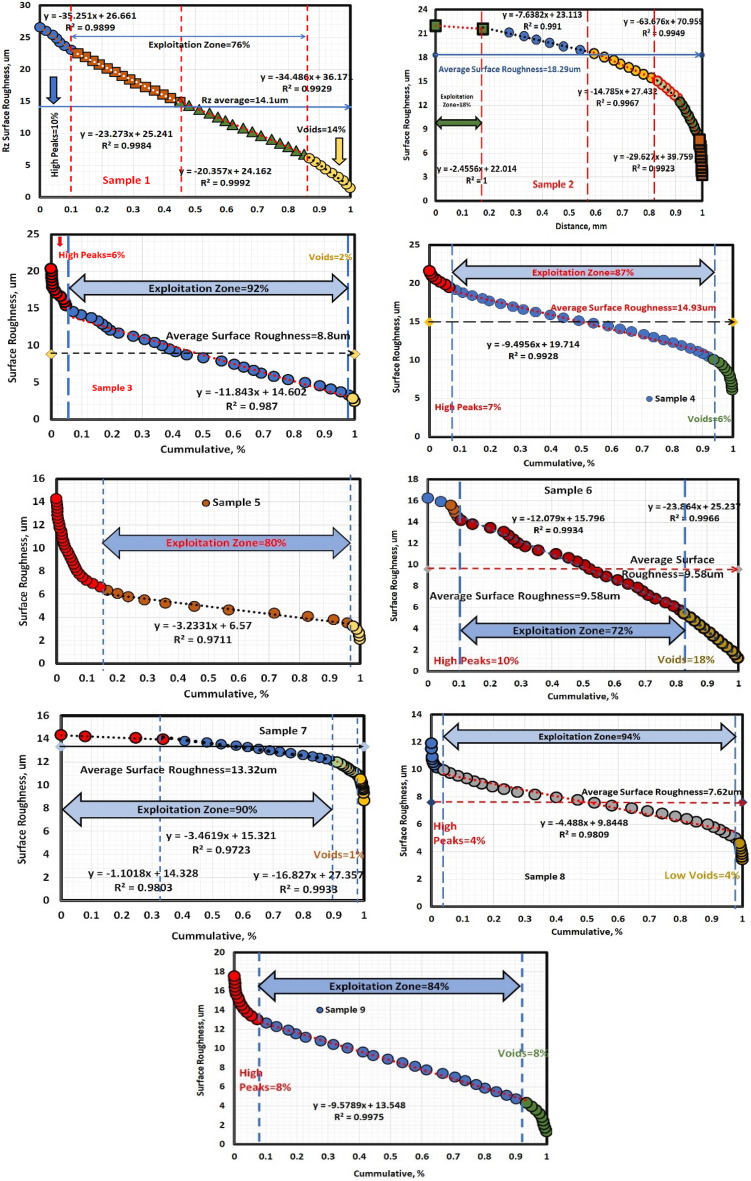
Abbott fire stone curves of different samples.

Figures 10 show the Abbott Firestone curves for various sample conditions. The curves can be divided into three zones:

- **Zone I:** The high peak zone, which approximately increases with increasing sliding speed in most conditions.
- **Zone II:** The exploitation zone, which approximately decreases with increasing sliding speed.
- **Zone III:** The voids zone.

In some cases, the voids zone may disappear, leaving only two zones: the high peak zone and the exploitation zone.

Figure 10 presents the Abbott Firestone curves for each sample, which indicate the exploitation zone (loading zone). The results show that all samples have a wide exploitation zone, with a minimum value of 76% or more. However, Sample 2 exhibits a small exploitation zone of only 18%, which suggests a higher risk of catastrophic failure.

### Interpretation of the results

The exploitation zone is the area of the surface that is most heavily loaded. This zone is also the most susceptible to wear and tear. The larger the exploitation zone, the more evenly the load is distributed across the surface, which can help to prevent catastrophic failure. The small exploitation zone in Sample 2 (0.01 MPa, 0.76 m/s) suggests that the load is not being distributed evenly across the surface. This could lead to localized wear and tear, which could eventually cause the sample to fail catastrophically.

### ANOVA of Abbott Firestone zones

The statistical design tool known as ANOVA allows for the differentiation of the individual impacts of the controlled variables. Finding statistically significant control factors is typically done using experimental data. Using DOE software and a response surface technique, the impacts of pressure (p) and velocity (V) on wear rate, peak height (Rz) and exploitation zone were statistically studied. Empirical Abbott Firestone models were then developed based on these effects.

**Figure 11:**
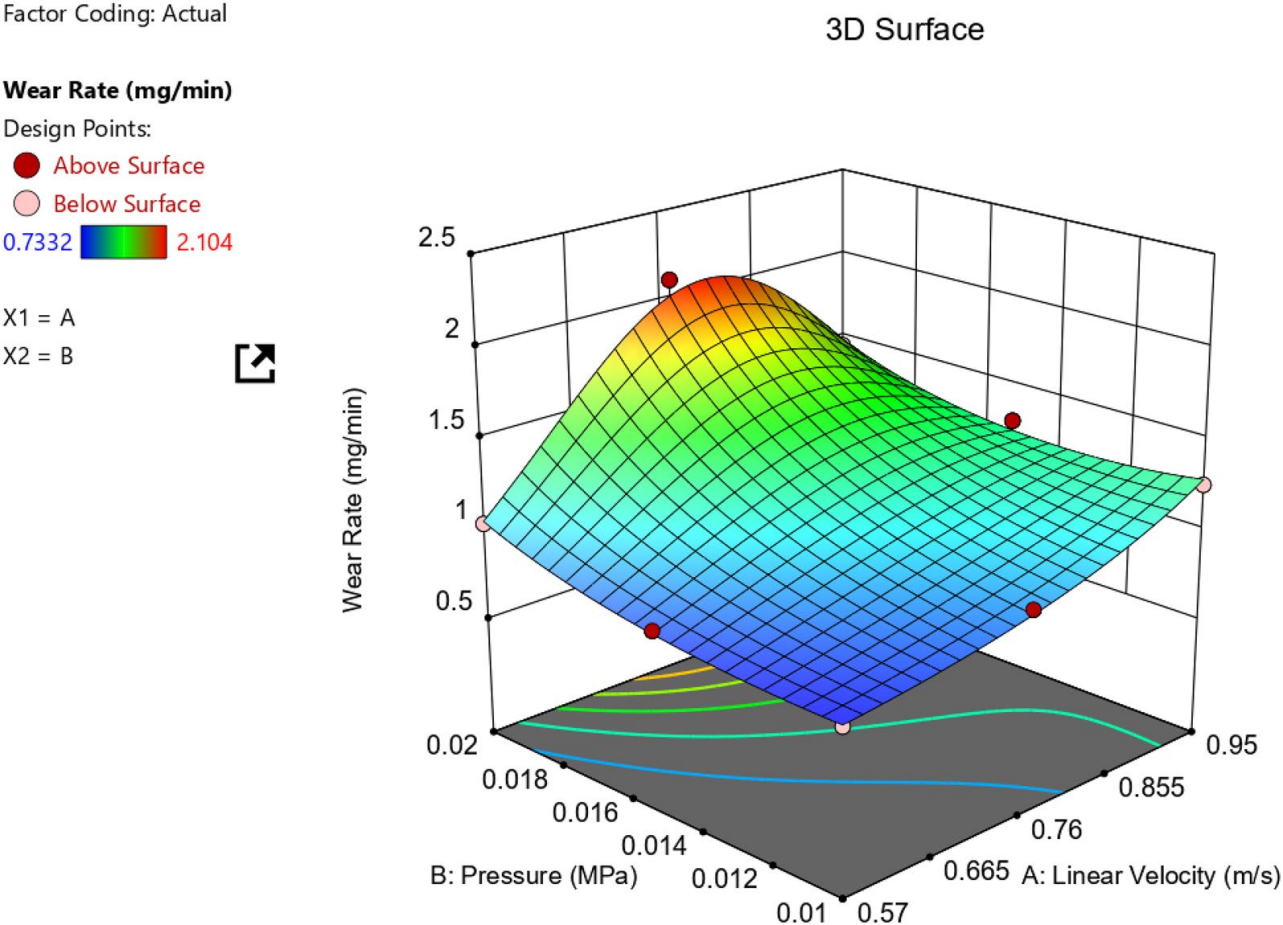
Relationship between different velocities and pressure on the wear rate.

To investigate the relationship between tribological parameters (pressure and velocity) and surface roughness, an experimental design approach was employed to evaluate the wear behavior. Figure 11 illustrates the effect of different pressure and velocity combinations on the wear rate (mg/min). The results show that the wear rate generally increases with increasing pressure and velocity, except for a notable tip at medium velocity and maximum pressure.

**Figure 12:**
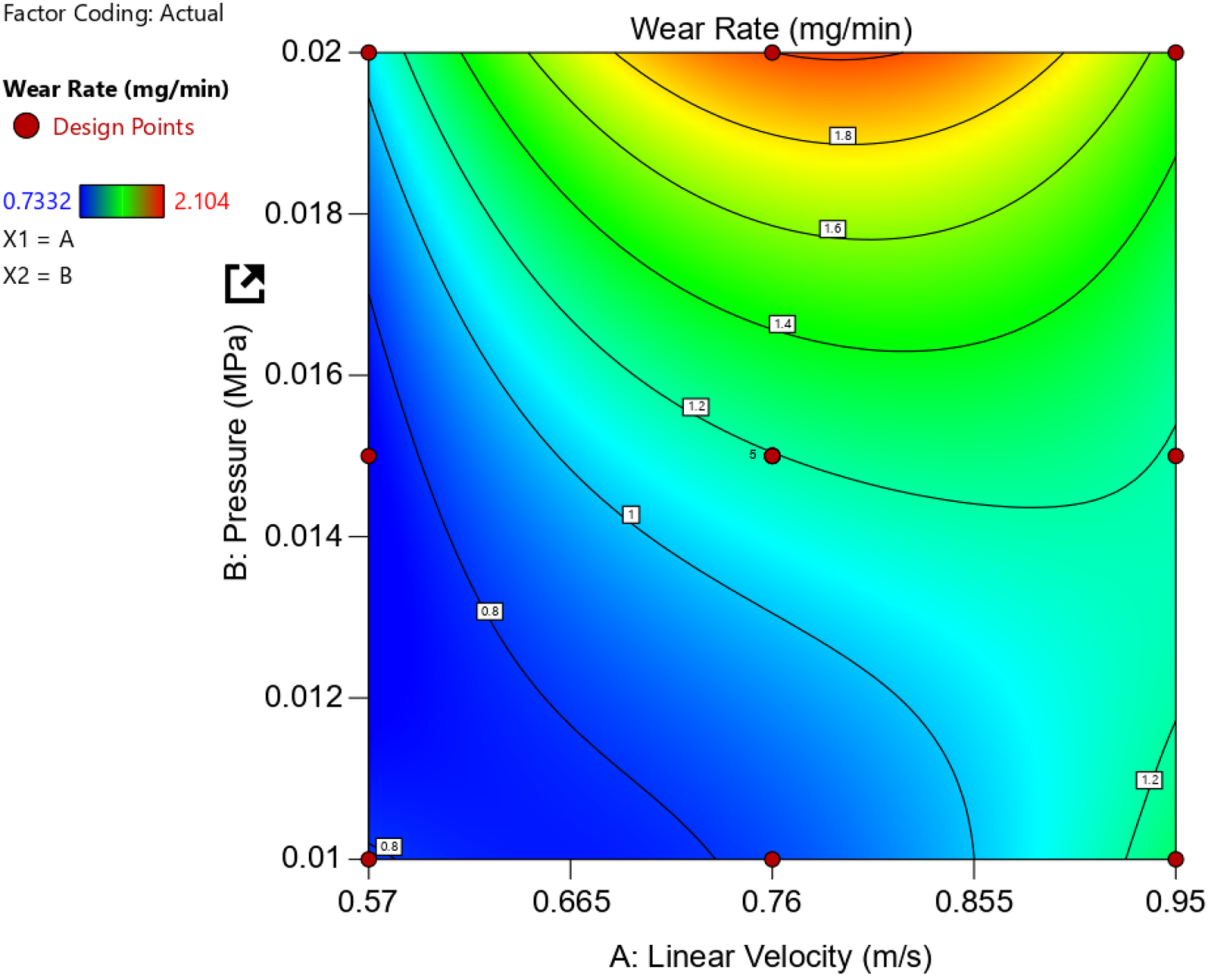
Contour relationship between linear velocity, pressure, and wear rate in mg/min.

It seems clear that the critical wear rate is at middle linear Velocity (0.76 m/s) and relatively high pressure (0.02 MPa). To deduce any wear rate value among the nine points of Table 1, another plot should be constructed (Contour Plot), as seen in Fig. 12. ANOVA results concerning wear rate response for AZ61Mg alloy are listed in Table 2.

**Table 2:** ANOVA results of reduced cubic model (wear rate is a response) for AZ61Mg alloy.

Source	Sum of squares	df	Mean square	F-value	p-value	
Model	0.1692	6	0.0282	109.27	< 0.0001	Significant
A-Linear velocity	0.0709	1	0.0709	274.87	< 0.0001	
B-pressure	0.0596	1	0.0596	230.90	< 0.0001	
AB	0.0037	1	0.0037	14.45	0.0090	
A^2^	0.0188	1	0.0188	72.93	0.0001	
B^2^	0.0061	1	0.0061	23.65	0.0028	
A^2^B	0.0163	1	0.0163	63.29	0.0002	
Residual	0.0015	6	0.0003			
Lack of fit	0.0015	2	0.0008			
Pure error	0.0000	4	0.0000			
Cor total	0.1707	12				
Std. dev	0.0161		R^2^		0.9909	
Mean	0.9415		Adjusted R^2^		0.9819	
C.V. %	1.71		Predicted R^2^		0.7831	
			Adeq precision		38.7121	
Factor coding is **c**oded			Sum of squares is Type III—Partial			

**Figure 13:**
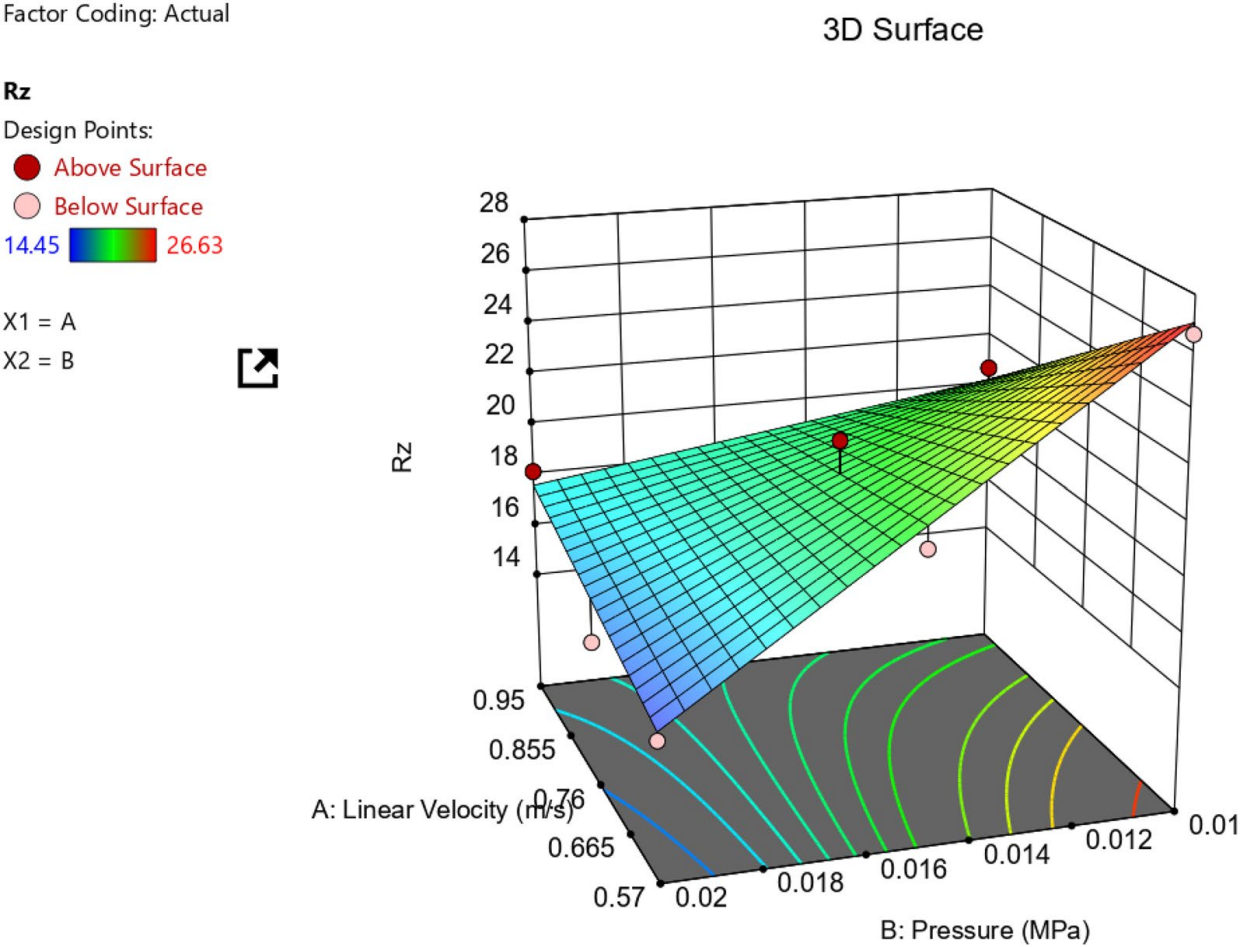
Surface roughness parameter Rz.

Figure 13 describes effect of both pressure and Velocity on surface roughness parameter (Rz). Also, ANOVA results for Rz response for AZ61Mg alloy are recorded in Table 3.

**Table 3:** ANOVA results of 2FI model (Rz is a response) for AZ61Mg alloy.

Source	Sum of squares	df	Mean square	F-value	p-value	
Model	112.15	3	37.38	15.87	0.0006	significant
A-Linear velocity	5.94	1	5.94	2.52	0.1468	
B-Pressure	83.03	1	83.03	35.25	0.0002	
AB	23.18	1	23.18	9.84	0.0120	
Residual	21.20	9	2.36			
Lack of fit	21.20	5	4.24			
Pure error	0.0000	4	0.0000			
Cor total	133.36	12				
Std. dev	1.53		R^2^		0.8410	
Mean	19.91		Adjusted R^2^		0.7880	
C.V. %	7.71		Predicted R^2^		0.7141	
			Adeq precision		14.3945	
Factor coding is coded			Sum of squares is Type III—Partial			

It seems clear that pressure (at low Velocity) is the dominant parameter increasing (Rz). While at high velocity, pressure has a slight effect.

**Figure 14:**
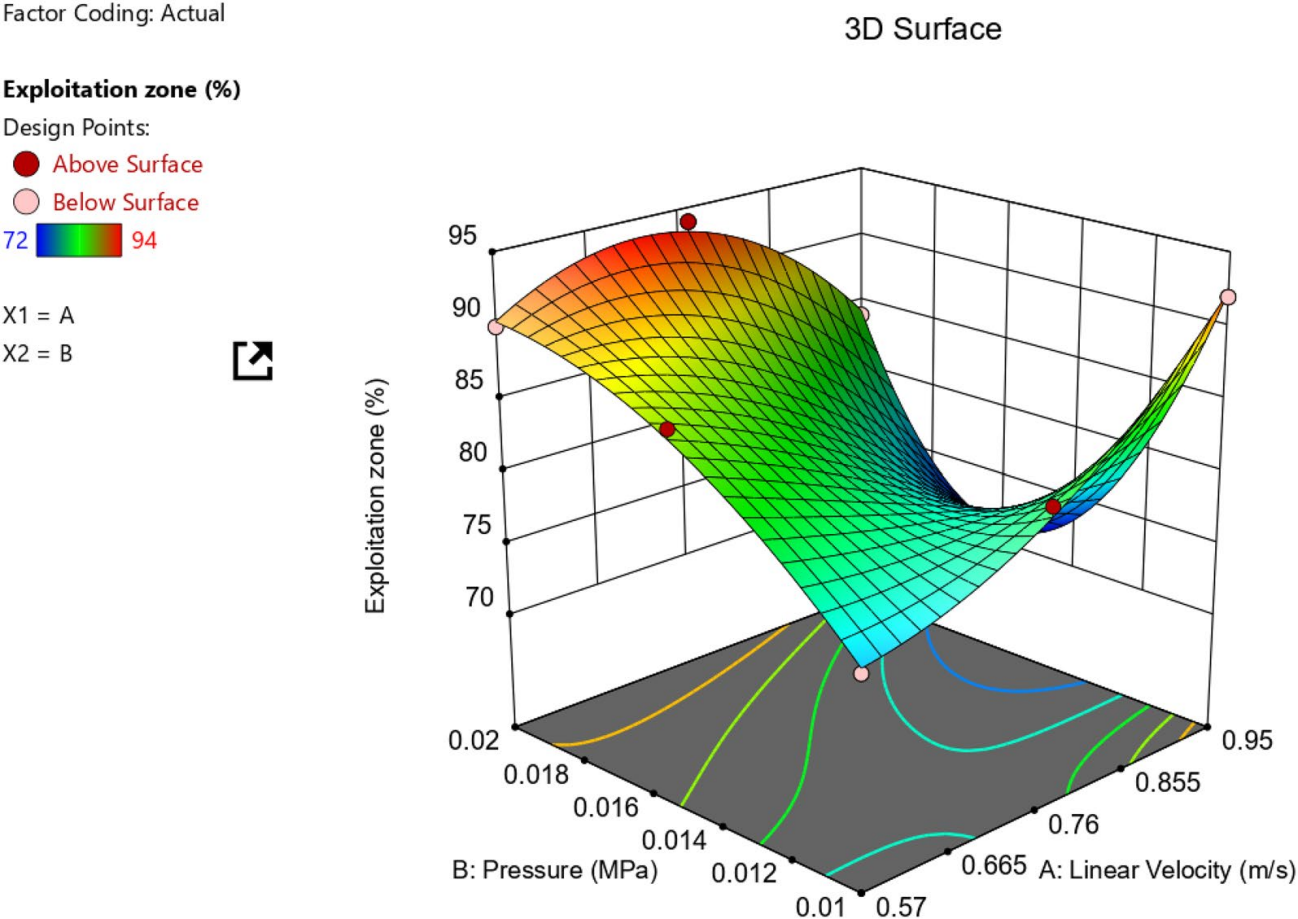
Effect of tribological parameters (velocities and pressures) on exploitation zone.

Figure 14 clarifies the effect of tribological parameters (velocities and pressures) on Exploitation zone of the worn surface. ANOVA results for exploitation zone response for AZ61Mg alloy are also shown in Table 4.

**Table 4:** ANOVA results of reduced cubic model (exploitation zone is a response) for AZ61Mg alloy.

Source	Sum of squares	df	Mean square	F-value	p-value	
Model	480.93	7	68.70	124.53	< 0.0001	Significant
A-Linear velocity	112.50	1	112.50	203.91	< 0.0001	
B-Pressure	72.00	1	72.00	130.50	< 0.0001	
AB	121.00	1	121.00	219.31	< 0.0001	
A^2^	5.93	1	5.93	10.75	0.0220	
B^2^	136.67	1	136.67	247.71	< 0.0001	
A^2^B	27.00	1	27.00	48.94	0.0009	
AB^2^	133.33	1	133.33	241.67	< 0.0001	
Residual	2.76	5	0.5517			
Lack of fit	2.76	1	2.76			
Pure error	0.0000	4	0.0000			
Cor total	483.69	12				
Std. dev	0.7428		R^2^		0.9943	
Mean	82.85		Adjusted R^2^		0.9863	
C.V. %	0.8966		Predicted R^2^		0.3373	
			Adeq precision		37.7562	
Factor coding is coded			Sum of squares is Type III—Partial			

It is obvious that exploitation zone highly increases at low pressure—high velocity or vice versa.

The significance of the models for wear rate, surface roughness parameter Rz, and exploitation zone was evaluated using the Model F-value and P-value. The Model F-value is a measure of the overall fit of the model, while the P-value is a measure of the significance of each term in the model. The model for wear rate is significant, with a Model F-value of 109.27 and P-values less than 0.05 for all terms. The model terms A, B, AB, A2, B2, and A2B are all important in predicting wear rate. The model for Rz is also significant, with a Model F-value of 15.87 and P-values less than 0.05 for the terms B and AB.

The model for the exploitation zone is the most significant, with a Model F-value of 124.53 and P-values less than 0.05 for all terms.

The Predicted R^2^ and Adjusted R^2^ values for all three parameters are reasonably close, indicating that the models are well-fitting. The Adeq Precision values for all three parameters are also strong, indicating that the signal-to-noise ratio is sufficient.

The empirical equations for all three parameters can be used to predict the response for specific levels of each factor. However, the equation for exploitation zone should not be used to estimate the relative importance of each factor, as the coefficients are scaled to account for the units of each factor, and the intercept is not in the middle of the design space.

The following table summarizes the results of the model evaluation:

**Table:** 

Parameter	Model F-value	P-value	Predicted R^2^	Adjusted R^2^	Adeq precision
Wear rate	109.27	< 0.05	0.7831	0.9819	38.712
Rz	15.87	< 0.05	0.7141	0.7880	14.394
Exploitation zone	124.53	< 0.05	0.3373	0.9863	37.756

In summary, the models for wear rate, Rz, and exploitation zone are all significant and well-fitting. The empirical equations for these models can be used to predict the response for specific levels of each factor.

## Conclusions

This study investigated the worn surface topography and mathematical modeling of AZ61Mg alloy using RSM. The following conclusions can be drawn from the results of the experiments and modeling:

1. AZ61Mg alloy has high plasticity due to its strong (0001) plane, which results in abundant plastic flow lines. However, complex precipitates (Mg17Al12) can make the alloy vulnerable to a transition from an adhesive to an abrasive wear mode.
2. At moderate velocity and high pressure, complex precipitates cause resonance phenomena, which lead to a tipping point.
3. When linear velocity and pressure increase, the wear rate also increases. However, the wear parameter Rz exhibits different trends under high and low pressure. Under high pressure, Rz only slightly increases with increasing linear velocity, while it significantly increases under low pressure. Conversely, under low pressure, Rz moderately decreases with increasing linear velocity.
4. At low pressure, the exploitation zone is an important factor in resisting material failure. The exploitation zone increases notably as linear velocity increases. Conversely, at low linear velocity, the same effect is achieved by increasing pressure. However, when high velocity is reached, exploitation values begin to drop dramatically as pressure dwindles, ultimately reaching a tipping point beyond which the values increase again at the same rate as that of the prior decrease.

Overall, understanding the mechanical behavior of AZ61Mg alloy is challenging. Analyzing its wear resistance and failure characteristics requires considering various factors, such as precipitate morphology, pressure, and velocity. This complexity makes AZ61Mg alloy a unique material with potential applications in a variety of fields.

## Data Availability

The datasets used and/or analyzed during the current study are available from the corresponding author upon reasonable request.

## References

[1] Shan Z, Zhang Y, Wang B, Zhang Q, Fan J (2022). Microstructural evolution and precipitate behavior of an AZ61 alloy plate processed with ECAP and electropulsing treatment. J. Mater. Res. Technol..

[2] Zagórski I, Korpysa J (2020). Surface quality assessment after milling AZ91D magnesium alloy using PCD tool. Materials.

[3] Yu Wu H, Chen Yang J, Jun Zhu F, Cheng Liu H (2012). Hot deformation characteristics of as-cast and homogenized AZ61 Mg alloys under compression. Mater. Sci. Eng. A.

[4] Elshaer RN, El-Fawakhry MK, Mattar T, Farahat AIZ (2022). Mathematical modeling of wear behavior and Abbott Firestone zones of 0.16C steel using response surface methodology. Sci. Rep..

[5] Zhang Z (2021). Toward the development of Mg alloys with simultaneously improved strength and ductility by refining grain size via the deformation process. Int. J. Miner. Metal. Mater..

[6] Wu HY, Yang JC, Liao JH, Zhu FJ (2012). Dynamic behavior of extruded AZ61 Mg alloy during hot compression. Mater. Sci. Eng. A.

[7] Tomescu, L., Ripa, M., & Georgescu, C. Analysing Abbott curve for composites with polymeric matrix and fibbers.

[8] Torrance AA (1997). A simple datum for measurement of the Abbott curve of a profile and its first derivative. Tribol. Int..

[9] Sosa M, Björklund S, Sellgren U, Olofsson U (2015). In situ surface characterization of running-in of involute gears. Wear.

[10] Sosa M, Sellgren U, Björklund S, Olofsson U (2016). In situ running-in analysis of ground gears. Wear.

[11] Affatato S, Ruggiero A, De Mattia JS, Taddei P (2016). Does metal transfer affect the tribological behaviour of femoral heads? Roughness and phase transformation analyses on retrieved zirconia and Biolox ® Delta composites. Compos. Part B.

[12] Mathia TG, Pawlus P, Wieczorowski M (2011). Recent trends in surface metrology. Wear.

[13] Bruzzone AAG, Costa HL, Lonardo PM, Lucca DA (2008). Advances in engineered surfaces for functional performance. CIRP Ann. Manuf. Technol..

[14] Karaa F (2023). Effect of cryogenic treatment on wear behavior of Sleipner cold work tool steel | Enhanced Reader. Tribol. Int..

[15] Nas E, O. O. Onur¨ozbek, F. Bayraktar, and F. Kara,  (2021). Experimental and statistical investigation of machinability of AISI D2 steel using electroerosion machining method in different machining parameters. Adv. Mater. Sci. Eng..

[16] Manoj IV, Soni H, Narendranath S, Mashinini PM, Kara F (2022). Examination of machining parameters and prediction of cutting velocity and surface roughness using RSM and ANN using WEDM of Altemp HX. Adv. Mater. Sci. Eng..

[17] Razzaq AM, Majid DL, Ishak MR, Muwafaq Basheer U (2020). Mathematical modeling and analysis of tribological properties of AA6063 aluminum alloy reinforced with fly ash by using response surface methodology. Crystals (Basel).

[18] Abdelmoneim A, Elshaer RN, El-Shennawy M, Sobh AS (2023). Modeling of wear resistance for TC21 Ti-alloy using response surface methodology.

[19] Chauhan SR, Dass K (2013). Dry sliding wear behaviour of titanium (Grade 5) alloy by using response surface methodology.

[20] S. Meddah *et al.* (2019) Prediction of the friction coefficient of 13Cr5Ni2Mo steel using experiments plans-study of wear behavior. In *Proceedings of the International Conference on Industrial Engineering and Operations Management Pilsen, Czech Republic, July 23–26,* (2019).

